# Sorcin promotes migration in cancer and regulates the EGF-dependent EGFR signaling pathways

**DOI:** 10.1007/s00018-023-04850-4

**Published:** 2023-07-13

**Authors:** Claudia Tito, Ilaria Genovese, Flavia Giamogante, Anna Benedetti, Selenia Miglietta, Lucia Barazzuol, Loredana Cristiano, Alessia Iaiza, Sabatino Carolini, Luciana De Angelis, Silvia Masciarelli, Stefania Annarita Nottola, Giuseppe Familiari, Vincenzo Petrozza, Mattia Lauriola, Luca Tamagnone, Andrea Ilari, Tito Calì, Hector H. Valdivia, Carmen R. Valdivia, Gianni Colotti, Francesco Fazi

**Affiliations:** 1 Section of Histology and Medical Embryology, Department of Anatomical, Histological, Forensic & Orthopaedic Sciences, Sapienza University of Rome, Via A. Scarpa, 14-16, 00161 Rome, Italy; 2 Institute of Molecular Biology and Pathology, Italian National Research Council, IBPM-CNR, P.le A. Moro 5, 00185 Rome, Italy; 3 Department of Biomedical Sciences, University of Padova, Padua, Italy; 4 Section of Human Anatomy, Department of Anatomical, Histological, Forensic & Orthopaedic Sciences, Sapienza University of Rome, Rome, Italy; 5 Department of Life, Health and Environmental Sciences, University of L’Aquila, L’Aquila, Italy; 6 Department of Medico-Surgical Sciences and Biotechnologies, Sapienza University of Rome, Latina, Italy; 7 Department of Experimental, Diagnostic and Specialty Medicine (DIMES), University of Bologna, Bologna, Italy; 8 Department of Life Science and Public Health, Histology and Embryology Unit - Catholic University of the Sacred Hearth, Fondazione Policlinico Gemelli - IRCCS, Rome, Italy; 9 Department of Medicine, Cardiovascular Research Center, University of Wisconsin, Madison, WI USA

**Keywords:** EGF, EGFR, Sorcin, Cancer, Calcium, Signaling, Migration, Invasion, Epithelial-to-mesenchymal transition

## Abstract

The epidermal growth factor receptor (EGFR) is one of the main tumor drivers and is an important therapeutic target for many cancers. Calcium is important in EGFR signaling pathways. Sorcin is one of the most important calcium sensor proteins, overexpressed in many tumors, that promotes cell proliferation, migration, invasion, epithelial-to-mesenchymal transition, malignant progression and resistance to chemotherapeutic drugs. The present work elucidates a functional mechanism that links calcium homeostasis to EGFR signaling in cancer. Sorcin and EGFR expression are significantly correlated and associated with reduced overall survival in cancer patients. Mechanistically, Sorcin directly binds EGFR protein in a calcium-dependent fashion and regulates calcium (dys)homeostasis linked to EGF-dependent EGFR signaling. Moreover, Sorcin controls EGFR proteostasis and signaling and increases its phosphorylation, leading to increased EGF-dependent migration and invasion. Of note, silencing of Sorcin cooperates with EGFR inhibitors in the regulation of migration, highlighting calcium signaling pathway as an exploitable target to enhance the effectiveness of EGFR-targeting therapies.

**Supplementary Information:**

The online version contains supplementary material available at 10.1007/s00018-023-04850-4.

## Introduction

The epidermal growth factor (EGF) receptor (EGFR, ErbB-1, HER1) is expressed in many tissues and has many functions during development, in healthy people and in several pathological conditions, including cancer [1, 2]. EGF binding to EGFR leads to receptor dimerization and autophosphorylation of many tyrosine residues in its C-terminal domain, allowing direct or indirect recruitment of proteins as growth factor receptor-bound protein 2 (Grb2), Gab1, the E3 ubiquitin ligase CBL, the phosphoinositide 3-kinase (PI3K) complex and Src kinase. Moreover, EGF binding to EGFR increased EGFR kinase activity and the activation of several signaling pathways, leading to control of cell survival and metabolism [3–6].

Activated EGFR may have different outcomes. At low (< 20 ng/mL) doses of EGF, ligand-bound EGFR is incorporated into clathrin-coated pits and is rapidly internalized to endosomes in a complex process named clathrin-mediated endocytosis (CME). This process leads to signal propagation, until EGFR is recycled back to the plasma membrane, or targeted to mitochondria, Golgi and/or nuclei [4, 6], or the signal is extinguished by lysosomal EGFR degradation, a process requiring receptor ubiquitination and its recognition by the endosomal sorting complex required for transport (ESCRT) machinery that drives EGFR sorting into intraluminal vesicles at multivesicular endosomes [1, 7, 8]; at higher EGF concentrations, another EGFR internalization pathway, i.e., non-clathrin-mediated endocytosis (NCE), can be activated [1, 2].

Although EGFR-dependent signaling is a normal feature of cells, allowing transduction of information from the microenvironment, EGFR is considered one of the main cancer drivers, with the PI3K-AKT axis and RAS-RAF-MEK-ERK axis representing the main EGFR signaling pathways for cancer cell proliferation, progression, migration and angiogenesis. Aberrant EGFR activation is frequently reported in many human tumors, and dysregulation of EGFR is associated with poor clinical outcomes such as reduced survival rate, metastasization and resistance to chemotherapy [3–5, 9, 10]. EGFR is an important therapeutic target for many cancers. Small molecule tyrosine kinase inhibitors (TKIs), as erlotinib, gefitinib and lapatinib, and EGFR-specific antibodies, as cetuximab and panitumumab, efficiently suppress ligand-stimulated EGFR kinase activity in clinical studies [11–13]. However, these drugs have little or no effect in most solid tumors, with the exception of non-small cell lung cancers (NSCLC) carrying activating mutations in EGFR, which initially respond to the TKIs but eventually develop resistance.

Calcium is important in EGFR internalization and in EGFR signaling pathways: EGF stimulation increases cytosolic calcium concentration, and Ca^2+^ perturbation impairs both EGFR CME and NCE [1, 14], with mechanisms linked with PLCγ1 and IP3. Further, calmodulin binds EGFR, increasing EGF-dependent EGFR activation [15–17]. Calcium is involved in EGFR proteostasis, since its removal is responsible for EGFR degradation and signaling downregulation [18]. However, the relationship between calcium homeostasis and EGFR signaling pathways is still poorly understood.

Sorcin (SOluble Resistance-related Calcium binding proteIN) is one of the most expressed calcium-binding proteins in many tissues and is overexpressed in many human cancers, including leukemias, lymphomas, breast, lung, gastric, ovarian, nasopharyngeal tumors, glioblastoma, astrocytoma, oligodendroglioma, adenocarcinoma and multidrug (MD)-resistant tumors, with respect to normal tissues [19, 20]. Sorcin promotes cell proliferation, migration, invasion, epithelial-to-mesenchymal transition (EMT), malignant progression and resistance to chemotherapeutic drugs, while its silencing increases cellular sensitivity to cisplatin and adriamycin and decreases cell proliferation, cell cycle blockage and apoptosis [19, 21, 22].

Sorcin is a key player in the regulation of several cellular functions: it is important in the ER calcium-dependent cascades, regulates size and Ca^2+^ content of the ER and ER vesicles, inhibiting RyR and activating SERCA [22–26]. Upon calcium binding, Sorcin undergoes large conformational changes, involving exposure of hydrophobic surfaces, that allow it to interact with calcium channels and exchangers like Ryanodine receptors (RyRs) and Sarco(endo)plasmic reticulum Ca^2+^-ATPase (SERCA). Sorcin decreases ER calcium release by inhibiting RyR and increases calcium entry by activating SERCA, thus increasing Ca^2+^ accumulation in the ER (and mitochondria), preventing ER stress and the UPR. Further, Sorcin regulates Ca^2+^ homeostasis also by regulating voltage-gated calcium channels (VGCCs), the plasma membrane Ca^2+^-ATPase (PMCA) and the Na^+^- Ca^2+^ exchanger (NCX) and colocalizes with N-methyl-D-aspartate receptors (NMDARs) and the IP3 receptor [26–32]. Sorcin regulates many proteins associated with tumorigenesis including NF-κB, STAT3, AKT, ERK1/2, IP3R, VEGF, MMPs and caspases and regulates apoptosis, as its silencing results in increased levels of proapoptotic genes, results in major mitosis and cytokinesis defects, blocks cell cycle progression in mitosis, increases the formation of rounded, polynuclear cells, induces apoptosis and cell death [33–40]. Indeed, the overexpression of Sorcin has been associated with several cancer cell types, especially in those resistant to chemotherapeutic treatments [41–51]. Sorcin can be considered an oncogene and a marker of multidrug resistance (MDR), since its overexpression confers MDR in cancer cell line models, while its silencing increases sensitivity to chemotherapeutic drugs [33, 38, 43, 45–47, 49, 52–54].

In the present study, we focused on the role of Sorcin in EGFR physiological and pathological processes, using the Sorcin knockout mouse model vs. the wild-type animal [55], and H1299 NSCLC cells expressing wt EGFR and high levels of Sorcin [56] as a model.

We first studied whether Sorcin interacts with EGFR and the molecular basis of such interaction. The role of Sorcin in the EGFR pathway upon treatment with low concentration EGF was also assessed by studying the effect of Sorcin silencing on EGFR signaling.

We therefore explored the role of Sorcin in migration, EMT and invasion, upon EGF stimulus, and the effect of Sorcin silencing in EGFR calcium signaling and regulation. Overall, we discovered important relationships between calcium homeostasis and EGFR signaling pathways and identified Sorcin as a key player of EGFR physiological and pathological roles, linked to Ca^2+^ dysregulation.

## Materials and methods

### Analysis of the TCGA PanCancer study

Sorcin (SRI) and EGFR genetic alterations and expression have been evaluated on the TCGA PanCancer study by interrogating the cBioPortal repository (https://www.cbioportal.org/). We have selected on the cBioPortal the Pan-cancer analysis of whole genomes [57] including 2,583 patients. Accession was done in January 2023.

Specifically, genetic alterations were visualized by analyzing the Oncoprint and the Co-occurrence functions. Expression of SRI in relation to SRI/EGFR copy numbers was evaluated using the Plots function. Evaluation of Pearson's correlations at transcript level was carried out considering the RNAseq datasets. Evaluation of Pearson's correlations at protein/phosphoprotein level was carried out on the TCGA datasets of glioblastoma multiforme (GBM), colorectal cancer (CRC), lung adenocarcinoma (LUAD) and high-grade serous ovarian cancer (OVCA), by selecting the Firehose Legacy TCGA study, for each pathology, and interrogating the proteomic RPPA dataset vs. SRI mRNA (RNAseq).

### Survival analysis

Analysis of the impact of SRI, EGFR and combined SRI/EGFR expressions on overall survival in various cancer types has been performed using the Kaplan–Meier plotter (https://kmplot.com) PanCancer function, by setting "auto select best cut-off" and considering all the available patients for each pathology. Mean expression of SRI and EGFR has been considered to evaluate these as a signature.

### Analysis of the cProSite study

Correlation of Sorcin and EGFR protein expression and phosphorylation was carried out using the Cancer Proteogenomic Data Analysis Site (cProSite), a web-based interactive platform that provides visualization of proteomic and phosphoproteomic analysis of the datasets of the National Cancer Institute’s Clinical Proteomic Tumor Analysis Consortium (CPTAC) and National Cancer Institute’s International Cancer Proteogenome Consortium (ICPC). Correlation of the expression levels of Sorcin and EGFR was calculated for 10 different types of tumors present in the database (1152 total patients; calculation of correlation in tumors, adjacent normal tissues, total correlation) and in all tumors overall. The average increase in the phosphorylation of Sorcin and EGFR in tumors vs. adjacent normal tissue was also calculated for the 7 tumors where such data were available. Accession was obtained in May 2023.

### Cell culture, transfection and treatment

Human non-small lung cancer cell line H1299 (ATCC, Manassas, VA, USA) and Calu-1 (ATCC, Manassas, VA, USA), and HeLa cervical cancer cell line (ATCC, Manassas, VA, USA) were cultured in DMEM (Gibco^®^ Thermo Fisher Scientific, Waltham, MA, USA) supplemented with 50 U/mL penicillin, 50 μg/ml streptomycin and 10% heat-inactivated FBS at 37 °C in incubator with humidified 5% CO_2_ atmosphere.

For Sorcin silencing, H1299, HeLa and Calu-1 cells were transiently transfected with Dicer-substrate short interfering RNAs (DsiRNAs) for Sorcin (IDT, Belgium) and negative control (NC) (#51-01-14-04, IDT, Belgium) at 500 pM for 48 h, using Lipofectamine RNAiMAX (Gibco® Thermo Fisher Scientific, Waltham, MA, USA) according to the manufacturer’s instructions. H1299 cells were also transfected with Dicer-substrate short interfering RNAs (DsiRNAs) for EGFR (IDT, Belgium).

For gene silencing, siRNAs sequences were: siSRI: 5´-GGCAUUGCUGGAGGAUACAAACCTT-3´, 5´-GACCGUAACGACCUCCUAUGUUUGGAA-3´; siEGFR: 5´-CCAUAAAUGCUACGAAUAU-3´, 5´-AUAUUCGUAGCAUUUAUGG-3´. After the transfection, cells were starved for 2 h in serum-free medium (SF) to examine the effect of EGF independently of other growth factors present in fetal bovine serum and to eliminate other variables which could interfere with EGF treatment. Then, cells were treated with: epidermal growth factor, EGF (Merck Millipore, Italy) at final concentration of 1 ng/mL and 5 ng/mL for the indicated time; erlotinib (#5083 Cell Signaling, Danvers, MA, USA), at final concentration of 5 μM for 24 h; thapsigargin (#T9033 Sigma-Aldrich, St. Louis, MO, USA), non-competitive inhibitor of the sarco/endoplasmic reticulum Ca^2+^ ATPase (SERCA), at final concentration of 100 nM for 6 h; insulin-like growth factor, IGF (Proteintech, USA) at final concentration of 1 ng/mL. For protein stability assays, cells were treated with 10 μg/ml cycloheximide (Sigma-Aldrich, St. Louis, MO, USA) for 6 h.

### Animal models

Sri^–/–^ knock-out (KO Sorcin) mice were provided by Héctor H. Valdivia and Carmen R. Valdivia (Department of Internal Medicine, Division of Cardiovascular Medicine, University of Michigan, Ann Arbor, MI 48109, USA). These mice were generated as described in the work of Chen et al. [56]. C57BL/6 wild-type mice were purchased from Jackson laboratory (Bar Harbor, ME, USA) and were housed in the Histology Department-accredited animal facility. All the procedures were approved by the Italian Ministry for Health and were conducted according to the US National Institutes of Health (NIH) guidelines (approval number 105/2017 PR).

### Lysate preparation and immunoblotting analysis

Cells were lysed in 2% SDS buffer (25 mM Tris–HCl pH 7.5, 100 mM NaCl, 3 mM EDTA, 7% glycerol) and fresh protease inhibitors. Lysates were sonicated for 15 s, centrifuged at 12,000 × rpm for 10 min to remove cell debris and then quantified in protein content with Bradford colorimetric assay (Thermo Fisher Scientific, Waltham, MA, USA) according to the manufacturer’s instructions. Western blotting was performed loading 30 μg of lysates and using the following primary antibodies: rabbit monoclonal EGFR (1:1000 in 5%BSA-T-TBS solution) (D38B1, #4267, Cell Signaling), rabbit monoclonal Phospho-EGF Receptor (1:1000 in 5%BSA-T-TBS solution) (Tyr1068, #3777 Cell Signaling, Danvers, MA, USA), rabbit monoclonal p44/42 MAPK (Erk1/2) (1:1000 in 5%BSA-T-TBS solution) (137F5, #4695 Cell Signaling, Danvers, MA, USA), rabbit monoclonal Phospho-ERK1/ERK2 (P44/P42 MAPK) (1:1000 in 5%BSA-T-TBS solution) (T202/Y204 # MAB-94112 Immunological Science, Italy), rabbit polyclonal CHD2 (1:1000 in 5%BSA-T-TBS solution) (#ab182013, Abcam), mouse monoclonal SLUG (A-7) (1:200 in 5%BSA-T-TBS solution) (#sc-166476 Santa Cruz Biotechnology, Heidelberg, Germany), mouse monoclonal SNAI1 (G-7) (1:200 in 5%BSA-T-TBS solution) (#sc-271977 Santa Cruz Biotechnology, Heidelberg, Germany), mouse monoclonal Sorcin (39-M) (1:200 in 5%BSA-T-TBS solution) (#sc-100859 Santa Cruz Biotechnology, Heidelberg, Germany), mouse monoclonal GAPDH (1:2000 in 5%BSA-T-TBS solution) (#TA802519, OriGene Technologies, Rockville, USA), mouse monoclonal α-Tubulin B-5–1-2 (1:3000 in 5%BSA-T-TBS solution) (#T5168, Sigma-Aldrich, Milan, Italy). As secondary antibodies were used goat anti-mouse (1:10,000 in 5% milk-T-TBS solution) and anti-rabbit (1:5000 in 5% milk-T-TBS solution or 5%BSA-T-TBS solution for phosphorylated protein) conjugated to horseradish peroxidase (Bethyl Laboratories, Montgomery, TX, USA). Protein signals were developed by ECL detection using a ChemiDoc-It Imaging System (UVP, Upland, CA) instrument.

### Total RNA extraction from cells, cDNA reverse transcriptase and RT-qPCR

Total RNA was extracted using the TRIzol RNA Isolation System (Invitrogen) according to manufacturer instructions. Reverse transcription to cDNA was performed with the High-Capacity RNA-to-cDNA Kit (Applied Biosystems), and cDNA was amplified using the SYBR™ Green PCR Master Mix (Thermo Fisher Scientific, Waltham, MA USA) on QuantStudio™ 7 Flex Real-Time PCR System, 384 well (Applied Biosystems). Quantification level of Sorcin was determined with the comparative 2 − ΔΔCt method, using H3 as control. All reactions were performed in duplicate. The following oligo sequences were used: H3 FW: 5´-GTGAAGAAACCTCATCGTTACAGGCCTGGT-3´; H3 RW: 5´-CTGCAAAGCACCAATAGCTGCACTCTGGAA-3´; SRI FW: 5´-GGCCACTCTGCAAGAAGGCA-3´; SRI RW: 5´TCCGGGAGCCCCTCCATACT-3´.

### Cell invasion assay

Cell invasion assay was performed using a 24-well plate with a non-coated 8-mm pore size filter in the insert chamber (BD Falcon, Franklin Lakes, NJ, USA) according to the manufacturer’s instructions. Each permeable support was covered with a diluted Matrigel matrix coating solution and was incubated at 37 °C for 2 h. After 48 h of transfection and 2 h of starvation in serum-free medium, 2.0 × 10^5^ H1299 cells were plated in the upper of each 24-well invasion chamber and then treated with EGF 1 ng/mL. To induce cellular invasion, in each bottom chamber was added 0.7 mL DMEM supplemented with 20% of FBS, used as chemoattractant.

At the end of the incubation, non-migrated cells present on the upper surface of the chamber were gently removed with a cotton swab. Then, migrated cells in the lower surface of the chamber were washed with PBS and nuclei were stained with Diff-Quik stain (Corning, Life Sciences Tewksbury, MA USA). For the quantification of cell invasion, stained cells were dissolved in an extraction buffer, and solutions were transferred to a 96-well culture plate for colorimetric reading of OD at 560 nm. The OD value represents the invasive ability.

### Wound-Healing assay

To perform wound-healing assay, cells 3.5 × 10^5^ cells/well were seeded into 6-well dishes and transfected with siRNA for Sorcin (siSRI) and Negative control (siNC). After 48 h, confluent cells were starved for 2 h in serum-free medium (SF) and a wound has been performed in the middle of the cell monolayer. Each well was photographed at 2.5 × magnification at time 0 and after 24 h of EGF 1 ng/mL, IGF 1 ng/mL and erlotinib 5 μM treatment, with a Nikon DS-Fi1 camera (Nikon Corporation, Tokyo, Japan), coupled with a Zeiss Axiovert optical microscope (Zeiss, Oberkochen, Germany). Quantification of wound-healing assay was calculated as percentage of wound closure: ((*A*_*t*0_ – *A*_*t*1_)/*A*_*t*0_) × 100) where *A*_*t*0_ is the initial wound area and *A*_*t*1_ is the wound area n hours after the initial scratch.

### FACS analysis

3.5 × 10^5^ cells/well were seeded into 6-well dishes and transfected with siRNA for Sorcin (siSRI) and Negative control (siNC). After 48 h, cells were starved for 2 h in serum-free medium (SF) and treated with EGF and erlotinib for 24 h. Cells were washed with ice-cold PBS, trypsinized and fixed for 10 min in ice-cold 4% paraformaldehyde. Then, cells were permeabilized with 0.01% Triton X-100 in PBS containing 1% BSA for 10 min and incubated with primary antibody rabbit monoclonal EGFR (1:200 in 1%BSA-PBS solution) (D38B1, #4267, Cell Signaling, Danvers, MA, USA) and rabbit monoclonal Phospho-EGF Receptor (1:200 in 1%BSA-PBS solution) (Tyr1068, #3777 Cell Signaling, Danvers, MA, USA), followed by incubation with Alexa fluor 488 (rabbit)-conjugated secondary antibodies (1:500 in 1%BSA-PBS solution) (Thermo Fisher Scientific, Waltham, MA, USA). Then, expression of proteins was quantified by acquired fluorescence on flow cytometry.

### Immunofluorescence analysis

3.5 × 10^5^ cells/well were seeded into 6-well dishes and transfected with siRNA for Sorcin (siSRI) and Negative control (siNC). After 48 h, cells were starved for 2 h in serum-free medium (SF) and treated with EGF for 24 h. Cells were fixed with 4% formaldehyde in PBS for 10 min at room temperature (RT) and permeabilized with 0.01% Triton X-100 in PBS for 5 min. Then, cells were incubated with rhodamine phalloidin (Thermo Fisher Scientific, Waltham, MA USA) diluted in PBS for 30 min, with primary antibody rabbit polyclonal KI67 (Abcam, Ab15580) followed by incubation with Alexa fluor 488 (rabbit)-conjugated secondary antibodies (Thermo Fisher Scientific, Waltham, MA USA). Then, nuclei were counterstained with Hoechst 33,342 (Life Technologies). Cells were mounted with Vectashield (DBA, Italy) and visualized under fluorescence confocal microscopy (Zeiss, Wetzlar, Germany).

For immunofluorescence in mice, lung organs were taken from four C57/BL6 mice and four KO Sorcin mice, covered in OCT mounting medium and frozen in liquid nitrogen-precooled isopentane. Ten-micrometer cryosections were fixed in 4% paraformaldehyde for 5´, washed in PSB and permeabilized in 0.01% Triton X-100 in PBS. Then, sections were blocked in 5% BSA-PBS solution for 1 h and incubated overnight at 4 °C with primary antibodies EGFR (1:50 in 1%BSA-PBS solution) (D38B1, #4267, Cell Signaling, Danvers, MA, USA) followed by incubation with Alexa fluor 488 (rabbit)-conjugated secondary antibodies (1:500 in 1%BSA-PBS solution) (Thermo Fisher Scientific, Waltham, MA, USA). Then, sections were counterstained with Hoechst 33,342 (Life Technologies), mounted with Vectashield (DBA, Italy) and visualized under fluorescence confocal microscopy (Zeiss, Wetzlar, Germany).

### Cell death and cell proliferation analysis

3.5 × 10^5^ cells/well were seeded into 6-well dishes and transfected with siRNA for Sorcin (siSRI) and Negative control (siNC). After 48 h, cells were starved for 2 h in serum-free medium (SF) and treated with EGF for 24 h. Cell death was evaluated by the propidium iodide exclusion assay and was analyzed by flow cytometry (CyAN ADP DAKO, Beckman Coulter, Brea, CA, USA). Cell proliferation was evaluated by Trypan blue exclusion assay and using a Dye eFluor 670 (Life Technologies), a red fluorescent dye that can be used to monitor individual cell division. Cells were resuspended at the 2 × final concentration in PBS, and Dye eFluor 670 was added at the 5uM final concentration. Cells were incubated 10 min at 37 °C in the dark and washed with 0.5 ml of complete medium for 3 times. 10^5^ cells were seeded in 12-well dish and transfected for 48 h with siRNA for Sorcin (siSRI) and Negative control (siNC). Then, cells were starved for 2 h in serum-free medium (SF), treated with EGF for 24 h and were analyzed by flow cytometry (CyAN ADP DAKO, Beckman Coulter, Brea, CA, USA).

### Surface plasmon resonance (SPR) experiments.

SPR experiments were carried out using a SensiQ Pioneer system (ICx Nomadics), essentially as in Genovese et al., 2020 [38]. The sensor chip (Ni–NTA) was activated chemically by a 35-μl injection of 0.1 M NiSO_4_ at a flow rate of 5 μl/min. Ligand, i.e., human EGFR C-terminal domain, was directionally immobilized on activated sensor chips via its N-terminal His-tag. The immobilization level of EGFR was 200 RU. Wt Sorcin and Sorcin W105G analytes were dissolved in buffer 20 mM Hepes pH 7.4, 150 mM NaCl + 0.005% surfactant P20 (HBS-P buffer) (± 100 μM CaCl_2_) to a concentration of 10 μM, automatically further diluted in HBS-P and injected on the sensor chip, at the following concentrations: 312 nM, 625 nM, 1.25 μM, 2.5 μM, 5 μM and 10 μM at a constant flow (nominal flow rate = 30 μl/min), in the presence of CaCl_2_ at 0 or 100 μM concentration. The increase in RU relative to baseline indicates complex formation; the plateau region represents the steady-state phase of the interaction (RUeq), whereas the decrease in RU after injection represents dissociation of Sorcin from immobilized EGFR after injection of buffer. Control experiments were performed in sensor chips treated as described above, in the absence of immobilized ligand. Regeneration procedures are based on two long (2000s and 500 s) injections of buffer, separated by a brief (5 s) injection of 5 mM NaOH. Kinetic evaluation of the sensorgrams was obtained using the SensiQ Qdat program and full fitting with 1, 2 and 3 sites.

### Calcium measurements

Ca^2+^ measurements were taken by co-transfecting H1299 or HeLa cells in a six-well plate with 1 μg cytosolic (cytAEQ) aequorin along with 5 nM of either scramble siRNA or siRNA against Sorcin, using Lipofectamine 3000. Twenty-four hours post-transfection, cells were re-plated into a 96-well plate (PerkinElmer). Forty-eight hours post-transfection, CytAEQ was reconstituted by incubating cells for 1.5 h with 5 µM coelenterazine wt (Santa Cruz Biotech) in modified Krebs Ringer Buffer (KRB: 125 mM NaCl, 5 mM KCl, 400 mM KH_2_PO_4_, 1 mM MgSO_4_, 20 mM Hepes, pH 7.4) supplemented with 0.1% glucose at 37 °C. Luminescence measurements were taken using a PerkinElmer EnVision plate reader equipped with two injector units. After reconstitution, cells were washed with KRB (either with 1 mM Ca^2+^ or 0.5 mM Ca^2+^, depending on the experiment) and luminescence from each well was measured for 1/1.5 min. According to the experiment, 100 μM Histamine (Sigma) or (Carbachol 500 μM + His 100 μM + ATP 100 μM + Bradykinin 100 nM) or (100 ng/mL EGF + CPA 20 μM + EGTA 2 mM) (final concentrations in the wells) was injected to generate Ca^2+^ transients, and then a hypotonic, Ca^2+^-rich, digitonin-containing solution was added to discharge the remaining aequorin pool. Output data were analyzed and calibrated with a custom-made macro-enabled Excel workbook.

## Results

### Sorcin and EGFR genetic alterations, expression and phosphorylation are correlated in the TCGA PanCancer study and in the cProSite study

**Fig. 1:**
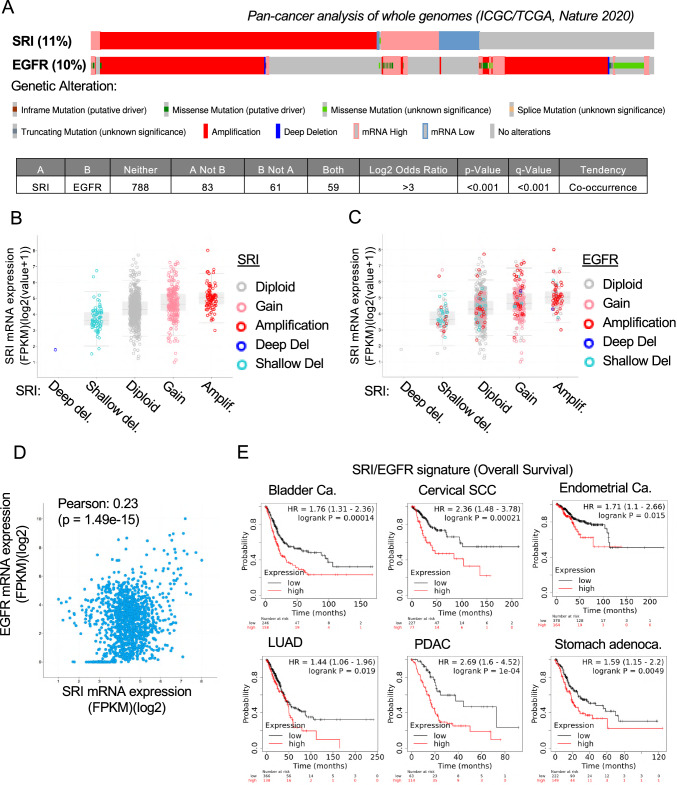
Sorcin and EGFR copy number alterations and expression are correlated in the TCGA PanCancer study. **A** Oncoprint visualization of Sorcin (SRI) and EGFR genetic alterations in the PanCancer TCGA study [57] considering 2583 patients. Evaluation of co-occurrence is shown in the bottom table. **B** SRI mRNA expression in the groups of tumors characterized by different SRI copy number status, as indicated from the color code in right legend. **C** Same plot as in (**B**), but showing as described by the color code the distribution of EGFR copy number. **D** Pearson's correlation analysis of EGFR and SRI mRNA expression in the PanCancer TCGA study. **E** Kaplan–Meier analysis of the impact of EGFR/SRI signature expression (RNAseq) on overall survival in the indicated tumor types: high level of expression of SRI and EGFR, considered as a signature (red), compared to low expression (black)

Analysis of genetic alterations occurring at Sorcin (SRI) and EGFR loci in the TCGA PanCancer study (cBioPortal) evidenced that these genes are altered in 8% and 10% of cases, respectively, and genetic alterations tend to co-occur (Fig. 1A). On this basis, we sought to explore whether SRI and EGFR copy number alteration were also correlated with their expression level. We first verified that Sorcin copy number alteration corresponded to an alteration of its mRNA expression (Fig. 1B). Specifically, this analysis evidenced that Sorcin expression paralleled SRI copy number content of tumors, such that tumors with Sorcin gain or amplification showed higher Sorcin expression level compared with tumors with diploid status or shallow deletion of the gene (Fig. 1B). We next explored the distribution of EGFR copy number in the groups characterized by different Sorcin copy number and expression. As shown in Fig. 1C, EGFR gains or amplifications are mainly localized in the groups with high Sorcin expression, e.g., those with gains or amplifications. A Pearson's correlation analysis of Sorcin and EGFR expression further confirmed a significant correlation between these two genes (Fig. 1D). Pearson's correlation analysis was also carried out to evaluate whether Sorcin expression also correlated with EGFR protein activation. As shown in Table 1, evaluation of proteomic data (RPPA) from TCGA profiling evidenced a significant correlation between Sorcin mRNA expression and EGFR pY1068 in various cancer types.

**Table 1:** Proteins/phosphoproteins correlated to SRI mRNA in TCGA-RPPA Datasets from the indicated cancer types (source: cBioPortal)

	GBM		CRC		LUAD		OVCA	
Protein	*R*	*p-Value*	*R*	*p-Value*	*R*	*p-Value*	*R*	*p-Value*
EGFR	0.445	3.25e-8	0.139	0.013	0.143	0.006		n.s
EGFR_PY1068	0.439	4.86e-8	0.202	0.0003	0.151	0.004	0.154	0.002
EGFR_PY1173	0.367	757e-7		n.s		n.s		n.s
ERBB2_PY1248	0.344	288e-6	0.122	0.031		n.s	0.145	0.003

*GBM* Glioblastoma Multiforme;* CRC* Colorectal Cancer;* LUAD* Lung Adenocarcinoma* OVCA* High-grade Serous Ovarian Cancer

We next evaluated whether the high expression of both Sorcin and EGFR is associated with survival. To this end we interrogated the Kaplan–Meier plotter database evaluating the PanCancer dataset. This analysis evidenced that high levels of expression of both Sorcin and EGFR, considered as a signature, is associated with reduced overall survival in various cancer types, compared to low expression (Fig. 1E). Sorcin and EGFR showed association with survival in the majority of the considered cancer types even when evaluated individually (Supplementary Fig. 1).

We also evaluated correlation of Sorcin and EGFR protein expression and phosphorylation, using the Cancer Proteogenomic Data Analysis Site (cProSite), a web-based interactive platform that provides visualization of proteomic and phosphoproteomic analysis of the datasets of the National Cancer Institute’s Clinical Proteomic Tumor Analysis Consortium (CPTAC) and National Cancer Institute’s International Cancer Proteogenome Consortium (ICPC). Expression of Sorcin and EGFR at protein level showed positive correlation in tumors, both overall (*R* =  + 0.12, by pooling all tumor data in the cProSite database) and in the majority of the considered cancer types when evaluated individually (positive correlation in 6 out of 7 tumors with at least 30 patients with data on abundance in tumors and adjacent normal tissue; Supplementary Fig. 2). In addition, we analyzed EGFR and Sorcin phosphorylation: the average increase of phosphorylation in tumors (phosphorylation in each tumor/phosphorylation in normal surrounding tissue ratio) was obtained for each type of tumor for EGFR and SRI. The correlation between the increase of phosphorylation in EGFR vs. SRI is very high, although limited by a low number of data for Sorcin (overall correlation >  + 0.9; Supplementary Fig. 2B).

Such positive correlations between genetic alterations, mRNA and protein expression, and phosphorylation in Sorcin and EGFR prompted us to study in vivo and in vitro whether Sorcin is involved in EGFR signaling.

### Sorcin knockout reduces EGFR in the bronchiolar region of mice lungs.

**Fig. 2:**
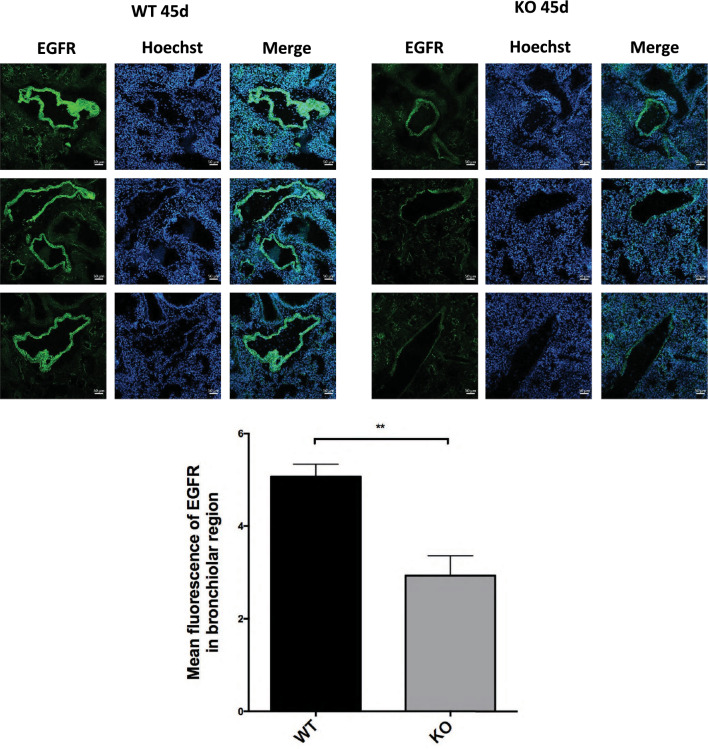
Sorcin knock-out reduces EGFR in the bronchiolar region of mice lungs. Confocal microscopy staining in lung tissues of adult (6 weeks old) C57/BL6 WT and KO Sorcin mice to evaluate the expression of EGFR protein (green). Scale bars, 50 µm. Quantification analysis of protein fluorescence intensity by ImageJ software plugin in the bronchiolar region. Error bars indicate means ± SEM. **p < 0.01 as determined by Student’s *t* test [analysis was performed in 10 lung’s section per 4 mice per group (C57/BL6 WT versus KO Sorcin mice)]

Confocal microscopy analysis of lung tissues of adult (6 weeks old) C57/BL6 WT and SRI^–/–^ knockout (KO Sorcin) mice was carried out to evaluate the expression of EGFR protein (Fig. 2 and Supplementary Fig. 3). Quantification analysis of protein fluorescence intensity was performed by the ImageJ software plugin in the bronchiolar region, in 10 lung sections per 4 mice per group (C57/BL6 WT versus KO Sorcin mice). The experiment showed that the expression of EGFR in Sorcin KO mice is strongly reduced with respect to WT mice, in the bronchiolar regions, which are mostly epithelial (*p* < 0.01).

### Sorcin regulates the EGF-dependent signaling

**Fig. 3:**
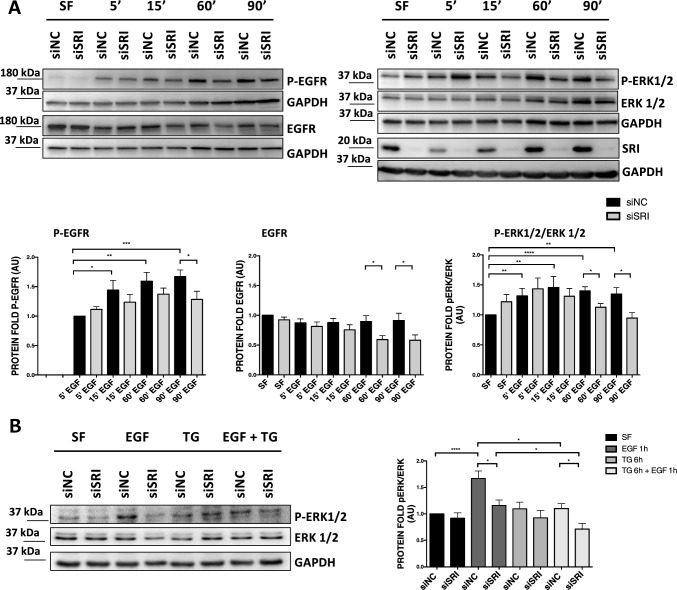
Role of Sorcin protein in the EGFR signaling pathway upon EGF stimulation. **A** H1299 cells were silenced for SRI protein (siSRI) for 48 h, starved for 2 h in serum-free medium (SF) and then treated or not with low doses of EGF (5 ng/mL) at the indicated time points. Western blot analysis showed Sorcin downregulation and total EGFR, EGFR and ERK 1/2 phosphorylation levels upon Sorcin silencing (siSRI) and EGF stimulation. Densitometry analysis is shown in the right graphs**.** Error bars indicate means ± SEM. *p < 0.05, **p < 0.01, ***p < 0.001, ****p < 0.0001 as determined by Student’s *t* test (*n* = 4). **B** H1299 cells were silenced for Sorcin (siSRI) for 48 h, then treated with thapsigargin (TG) [an inhibitor of sarco/endoplasmic reticulum Ca^2+^ ATPase (SERCA)] for 6 h and then with EGF for 1 h, after 2 h of starvation in serum-free medium (SF). Western blot analysis of p-ERK1/2 and ERK1/2 expression. Densitometry analysis is shown in the lower graph. Error bars indicate means ± SEM. *p < 0.05, ****p < 0.0001 as determined by Student’s *t* test (*n* = 3)

EGFR signaling is known to occur with a calcium-dependent mechanism [1, 14]. H1299 NSCLC cells were treated with low doses (5 ng/mL) of EGF to understand whether Sorcin, which regulates calcium homeostasis in the cells and in particular ion concentration in endoplasmic reticulum, modulates EGFR signaling. Since Sorcin is upregulated in H1299 cells, we transfected them with a specific siRNA against Sorcin (siSRI) and with the respective negative control (siNC). H1299 cells silenced for Sorcin protein (siSRI cells) for 48 h and then starved for 2 h in serum-free medium (SF) and were treated with low doses of EGF (5 ng/mL) for the indicated time points (Fig. 3A). We observed that EGFR phosphorylation is increased upon EGF treatment and that Sorcin silencing causes a significant decrease of both phosphorylated EGFR and total EGFR proteins (Fig. 3A). Moreover, we detected a significant decrease of ERK1/2 phosphorylation level at 60 min (*P* = 0.0026) and at 90 min (*P* = 0.0424) of EGF stimulation in Sorcin-silenced cells, suggesting that Sorcin impacts EGFR signaling (Fig. 3A).

### Calcium mishandling mimics the effect of Sorcin on EGF-dependent signaling in NSCLC cells

As described previously, Sorcin is a calcium-binding protein which regulates ER calcium concentration, activating SERCA and inhibiting RyR; Sorcin silencing determines inactivation of SERCA and increased RyR activity and consequently a reduction of ER calcium concentration [25–27]. We therefore investigated whether thapsigargin, an inhibitor of SERCA able to reduce ER calcium content, impacted on EGFR signaling, similarly to Sorcin. H1299 NSCLC control cells (siNC) or silenced for Sorcin protein (siSRI) for 48 h, were treated with thapsigargin (TG) for 6 h and then with EGF for 1 h, after starvation in serum-free medium (SF). We investigated the effect of TG treatment on EGFR downstream signaling. We evidenced that treatment with TG leads to a reduction of pERK 1/2 upon EGF treatment, and that silencing of Sorcin in the presence of TG treatment further reduces pERK 1/2 level (Fig. 3B).

### Sorcin regulates EGF-dependent cell migration and invasion

**Fig. 4:**
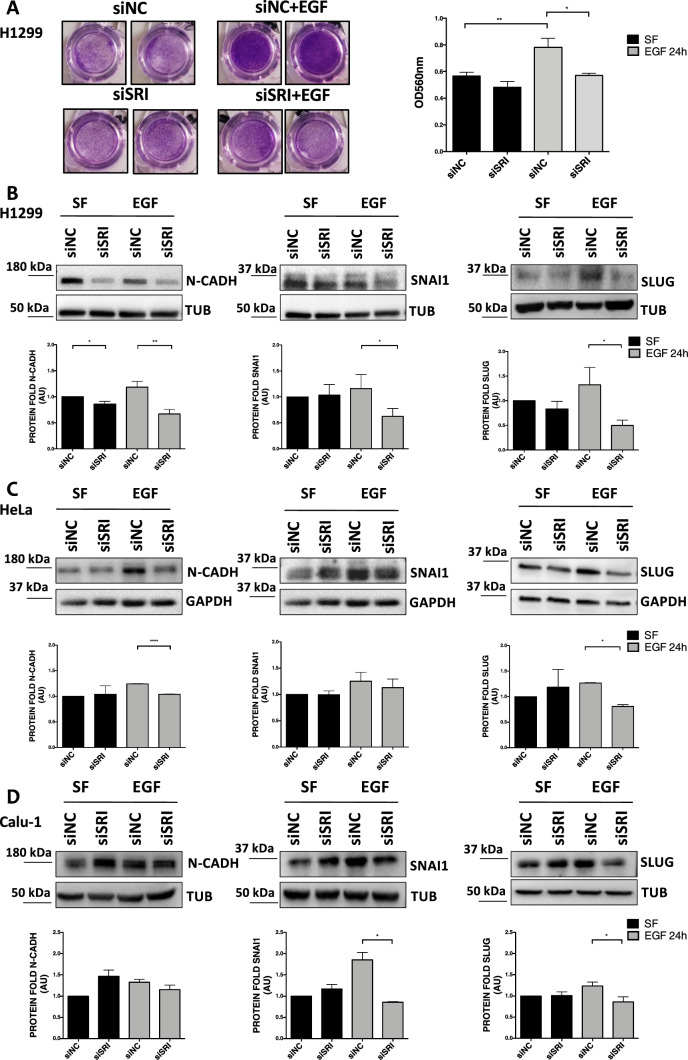
Involvement of Sorcin in the regulation of invasion and EMT program in H1299, HeLa and Calu-1 upon EGF stimulation. **A** Invasion was evaluated through Matrigel-coated transwell assay in H1299 cells cultured in serum-free medium (SF) (left panels) or in the presence of EGF (right panels) in control experiments (siNC, top panels) and upon silencing of Sorcin (siSRI, bottom panels). Crystal violet staining was measured at 590-nm absorbance. Error bars indicate means ± SEM. *p < 0.05 and **p < 0.01 as determined by Student’s *t* test (*n* = 3). **B–D** Western blot analysis of EMT proteins N-cadherin, Snai1 and Slug, upon 48 h of Sorcin silencing (siSRI), 2 h of starvation in serum-free medium (SF) and 24 h of EGF stimulation in H1299 (**B**), HeLa (**C**) and Calu-1 (**D**) cells. Densitometry analysis is shown in lower graphs**.** Error bars indicate means ± SEM. *p < 0.05, **p < 0.01 and ****p < 0.0001 as determined by Student’s *t* test (*n* = 3)

We therefore investigated the impact of Sorcin on EGF-dependent cell motility in H1299 and Calu-1 NSCLC and in HeLa cervical cancer cells. The EGFR protein, once activated by ligand binding, promotes the activation of a protein cascade that plays an important role in the mechanism of tumorigenesis, regulating cell proliferation, migration, differentiation and apoptosis [1–5]. Since Sorcin is overexpressed in different types of tumors and is involved in the calcium-linked regulation of pathways triggered by EGFR [35, 37], we investigated the role of Sorcin in EGF-dependent invasion of cancer cells. Specifically, we compared the cells in starved condition (SF) (serum-free medium that limits invasion, according to Inamura et al. [58]) without or with EGF stimulation. By invasion assay, we observed that silencing of Sorcin led to decreased invasive ability in H1299 cells, being this reduction statistically significant only in the presence of EGF stimulation (Fig. 4A). To support the cell motility, we also tested by Western blot analysis the levels of genes that are involved in the EMT process (such as N-cadherin, Snai1 and Slug) even though tumors can exhibit a partial EMT due to different expression of EMT markers that does not always correlate with the migratory potential and metastasization process [59, 60]. N-cadherin is a transmembrane, homophilic glycoprotein belonging to the calcium-dependent cell adhesion molecule family, that mediates homophilic interactions between adjacent cells; Snai1 and Slug are two well-characterized transcription factors which play an important role in the cellular invasion and metastasis. Increased expression of N-cadherin is a hallmark of EMT and endows tumor cells with enhanced migratory and invasive capacity [61]. Western blot analysis showed a statistically significant decrease of N-cadherin (– 45%, *P* = 0.0099), Snai1 (– 50%, *P* = 0.0139) and Slug (– 58%, *P* = 0.0318) proteins in Sorcin-silenced H1299 cells compared to respective negative control cells, only upon 24-h EGF stimulation (Fig. 4B). Similar trends were obtained also in HeLa cells (Fig. 4C) and in Calu-1 cells (Fig. 4D). Quantification of Sorcin transcript expression upon siRNA transfection in H1299, HeLa and Calu-1 cells (*P* < 0.001) is included in Supplementary Fig. 4A. Altogether, these results support the involvement of Sorcin protein in the regulation of invasion and EMT program through EGFR signaling activation.

Cancer cells migration and invasion of the surrounding environment is favored by the reorganization of the actin cytoskeleton and a polarized morphology with membrane cytoskeletal protein actin projection. Therefore, we performed a phalloidin immunofluorescence experiment to observe the structure and possible changes of actin cytoskeleton. Confocal analysis showed that H1299 NSCLC cells silenced for Sorcin, after 2 h of starvation in serum-free medium (SF) and 24 h of EGF treatment, are characterized by lower actin polymerization and a reduction of stress fiber formation compared to control siNC cells, in line with reduced cell invasion and migration ability following Sorcin silencing (Supplementary Fig. 4D). Moreover, quantification of fluorescence intensity of phalloidin showed a significant difference between negative control cells and Sorcin-depleted cells, after EGF stimulation (Supplementary Fig. 4C).

A wound-healing assay was therefore carried out in H1299 and Calu-1 NSCLC cells and in HeLa cells cultured in serum-free medium (SF) or in the presence of EGF, upon silencing of Sorcin and in control cells, to test the role of Sorcin in migration. Images were acquired at 0 h and 24 h of EGF treatment (1 ng/mL) alone and combined to treatment with erlotinib, an EGFR kinase activity inhibitor used in therapy of NSCLC and pancreatic cancer, to test the effect of Sorcin silencing in EGFR-dependent migration on H1299 cells. In this assay, we captured the images at the beginning (0 h), when we performed the scratch and then, after 24 h of treatments, to measure migration rate of the cells; quantification of wound-healing assay was calculated as percentage of wound closure.

**Fig. 5:**
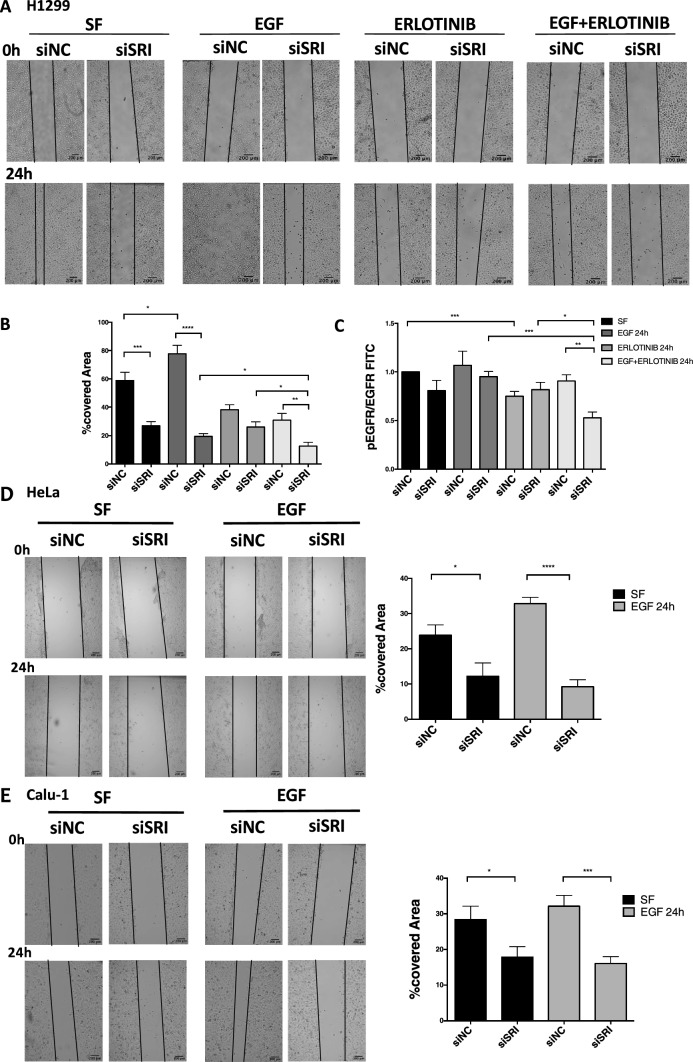
Role of Sorcin in cell migration after EGF and/or Erlotinib treatment. **A** Wound-healing assay in H1299 cells cultured in serum-free medium (SF) or in the presence of EGF and erlotinib, in control experiments (siNC) and upon silencing of Sorcin (siSRI). Images were acquired at 0 h and 24 h after each single and combined treatment. **B** Quantification of wound-healing assay calculated as percentage of wound closure: ((*A*_*t*0_ – A_t1_)/ *A*_*t*0_) × 100), where At_0_ is the initial wound area and A_t1_ is the wound area n hours after the initial scratch. Error bars indicate means ± SEM. *p < 0.05, **p < 0.01, ***p < 0.001 and ****p < 0.0001 as determined by Student’s *t* test (n = 3). Scale bars, 200 µm. **C** Flow cytometric analysis showed expression of total and phosphorylated EGFR upon Sorcin silencing and 24 h of EGF and/or erlotinib treatment. Error bars indicate means ± SEM. *p < 0.05, **p < 0.01, and ***p < 0.001 as determined by Student’s *t* test (*n* = 3). **D–E** Wound-healing assay in HeLa (**D**) and Calu-1 (**E)** cells cultured in serum-free medium (SF) or in the presence of EGF, upon silencing of Sorcin (siSRI). Images were acquired at 0 h and 24 h after EGF treatment (1 ng/mL). Quantification of wound-healing assay calculated as percentage of wound closure: ((*A*_*t*0_ – *A*_*t*1_)/*A*_*t*0_) × 100), where *A*_*t*0_ is the initial wound area and *A*_*t*1_ is the wound area n hours after the initial scratch. Error bars indicate means ± SEM. *p < 0.05, ***p < 0.001 and ****p < 0.0001 as determined by Student’s *t* test (*n* = 3). Scale bars, 200 µm

In H1299 cells, in serum-free medium condition (SF), Sorcin-silenced cells (siSRI) migrated at a slower rate (– 56%, *P* = 0.0006) compared to control cells (siNC) (Fig. 5A, B). Twenty-four hours after EGF treatment, which increases migration with respect to control (+ 32%, *P* = 0.0389), siSRI cells showed an even more reduced migration ability (– 75%, *P* =  < 0.0001) compared to negative control cells (Fig. 5A, B) and compared to siSRI cells cultured in serum-free medium (SF) (– 30%, *P* = 0.051). Similar results were obtained in HeLa and in Calu-1 cells (Fig. 5D, E). As we expected, erlotinib treatment reduced cell migration in siNC cells compared to negative control cells in serum-free medium (SF) and EGF stimulation (respectively, – 35%, *P* = 0.0220 and – 50%, *P* = 0.0002), indicating an effect on EGFR pathway activation. Of note, combination of erlotinib treatment and silencing of Sorcin further reduced the migration ability compared to the single experimental conditions of erlotinib and siSRI, in the presence of EGF (Fig. 5A, B).

To test erlotinib activity on EGFR protein expression, we quantified total and phosphorylated EGFR expression by flow cytometry. Quantification analysis showed that erlotinib led to a decrease of phosphorylated EGFR in negative control cells compared to serum free (SF) (Fig. 5C). Of note, combination of erlotinib treatment and silencing of Sorcin further reduced the phosphorylation of EGFR compared to the single experimental conditions of erlotinib and siSRI, in the presence of EGF (Fig. 5C). No significant effects of Sorcin silencing on cell proliferation and cell death, in the presence and absence of EGF, were observed (Supplementary Fig. 4B). According to these data, confocal and quantification analysis for KI67-cellular marker of proliferation (Supplementary Fig. 5A, B), and eFluor assay (Supplementary Fig. 5C) showed no changes in terms of proliferation between samples.

### Sorcin regulates EGFR levels and specifically acts on EGFR pathway

Sorcin and EGFR levels are co-regulated. In control H1299 cells, upon EGF stimulation, the levels of Sorcin are increased; conversely, in EGFR-silenced cells, levels of Sorcin are strongly decreased upon EGF stimulation. Levels of EGFR are also decreased upon EGF stimulation, in Sorcin-silenced cells (Supplementary Fig. 6A).

**Fig. 6:**
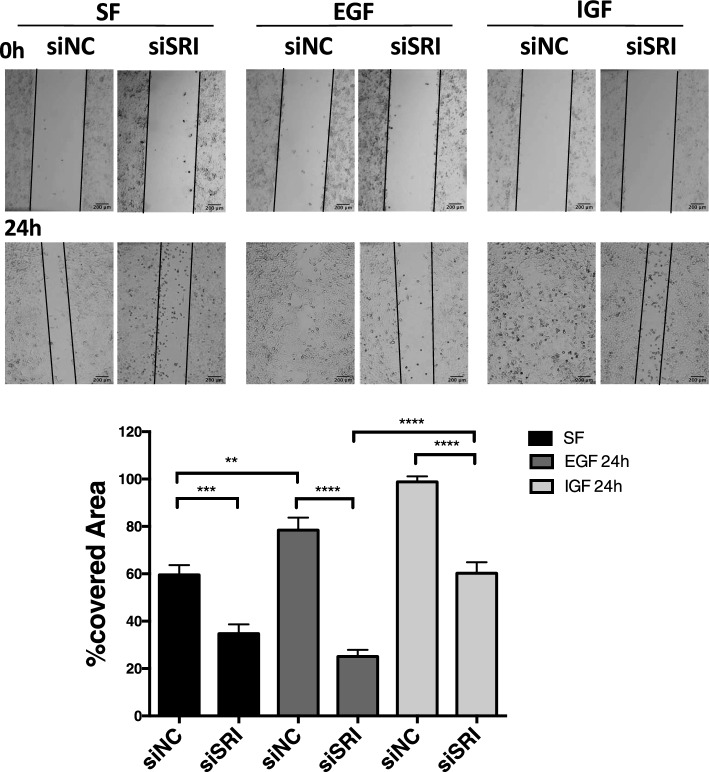
Role of Sorcin in cell migration after EGF and IGF treatment. Wound-healing assay in H1299 cells cultured in serum-free medium (SF) or in the presence of EGF and IGF, in control experiments (siNC) and upon silencing of Sorcin (siSRI). Images were acquired at 0 h and 24 h after each single treatment. Quantification of wound-healing assay calculated as percentage of wound closure: ((*A*_*t*0_ – *A*_*t*1_)/ *At*_0_) × 100), where *A*_*t*0_ is the initial wound area and *A*_*t*1_ is the wound area *n* hours after the initial scratch. Error bars indicate means ± SEM. **p < 0.01, ***p < 0.001 and ****p < 0.0001 as determined by Student’s *t* test (*n* = 3). Scale bars, 200 µm

Wound-healing assays show that Sorcin silencing decreases migration in serum-free conditions, upon EGF stimulation and upon IGF stimulation (Fig. 6). Migration is decreased by about 40% in serum-free conditions and upon IGF stimulation, and by about 70% by EGF stimulation. Part of the effect is possibly due to the alteration of calcium homeostasis dependent on Sorcin silencing (see below); however, the marked reduction of migration observed upon EGF stimulation in the absence of Sorcin further supports that Sorcin impacts the EGF-dependent EGFR pathway.

### Sorcin binds EGFR in a calcium-dependent fashion

**Fig. 7:**
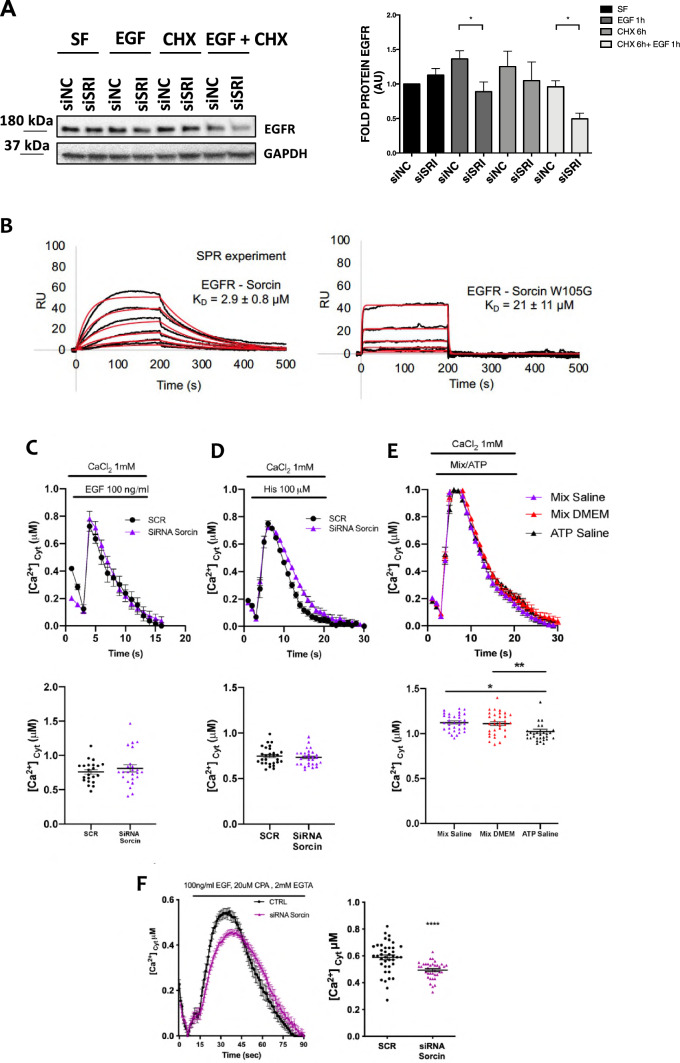
Sorcin binds EGFR and alters EGFR-dependent calcium signaling.** A** Western blot analysis of H1299 cells silenced for Sorcin (siSRI) for 48 h, treated with cycloheximide (CHX) for 6 h to inhibit EGFR synthesis and then treated with EGF for 1 h, after 2 h of starvation in serum-free medium (SF). Densitometry analysis is shown in the right graph**.** Error bars indicate means ± SEM. *p < 0.05 as determined by Student’s *t* test (*n* = 3). **B** SPR experiments showing the binding of wt Sorcin (left) and Sorcin W105G (right) to immobilized C-terminal intracellular domain of EGFR. Sorcin was injected at the following concentrations: 312 nM, 625 nM, 1.25 μM, 2.5 μM, 5 μM and 10 μM, at a constant flow (nominal flow rate = 30 μl/min), in the presence of CaCl_2_ at 100 μM concentration; kinetic evaluation of the sensorgrams was obtained using the SensiQ Qdat program and full fitting with 1 site (Langmuir interaction). EGFR binds wt Sorcin directly and with high affinity (*K*_D_ = 2.9 μM), while the affinity of EGFR for Sorcin W105G mutant is an order of magnitude lower (*K*_D_ = 21 μM). **C**–**F** Luminescence-based Ca^2+^ measurement using an organelle-targeted aequorin probe specific for the cytoplasm (cytAEQ) showing cytosolic Ca^2+^ transients of H1299 or HeLa cells transfected with either a non-specific control siRNA or a specific siRNA against Sorcin measured after their exposure to stimuli. Time-dependent luminescence measurements (top) and cytosolic Ca^2+^ measurements (bottom) were obtained. Error bars indicate means ± SEM. *p < 0.05; **p < 0.01; ****p < 0.0001, as determined by Student’s *t* test. **C** Measure of cytosolic Ca^2+^ transients of H1299 cells transfected with either a non-specific control siRNA (SCR) or a specific siRNA against Sorcin measured after their exposure to 100 ng/mL EGF. **D** Measure of cytosolic Ca^2+^ transients of H1299 cells transfected with either a non-specific control siRNA or a specific siRNA against Sorcin measured after their exposure to 100 μM Histamine. **E** Measure of cytosolic Ca^2+^ transients of H1299 cells transfected with either a non-specific control siRNA or a specific siRNA against Sorcin measured after their exposure to EGF in the presence of 2 mM EGTA, to prevent the influx of extracellular calcium, and 20 μM CPA, to inhibit SERCA pumps and the reuptake of calcium in the ER. **F** Measure of cytosolic Ca^2+^ transients of HeLa cells transfected with either a non-specific control siRNA or a specific siRNA against Sorcin, measured after their exposure to 100 ng/mL EGF

We also tested the effect of cycloheximide (CHX), the most common protein synthesis inhibitor, on EGFR in control and Sorcin-silenced H1299 cells. Oksvold et al. showed that the effect of CHX on EGFR is complex, since they observed CHX-dependent EGFR internalization in endosomes and EGFR signal inhibition, but not EGFR degradation [62]. We silenced Sorcin (siSRI) for 48 h, treated with CHX for 6 h, and then with EGF for 1 h, after 2 h of starvation in serum-free medium. Western blot analysis showed a reduction of total EGFR in siSRI cells compared to control cells upon cycloheximide treatment (– 49%, *P* = 0.0178), indicating that Sorcin silencing could decrease EGFR proteostasis (Fig. 7A).

We finally tested whether Sorcin binds EGFR. We carried out surface plasmon resonance experiments, to measure the physicochemical parameters of EGFR binding. We immobilized the C-terminal intracellular domain of EGFR and tested its interaction with wt Sorcin vs. Sorcin W105G, the latter being a Sorcin mutant bearing a single-residue mutation in the EF3 hand, that lowers its affinity for calcium and impairs Sorcin activation, the exposure of the EF3 hydrophobic pocket and targets binding [24, 25, 63]. In the presence of 100 μM calcium, EGFR binds wt Sorcin directly and with high affinity (*K*_D_ = 2.9 μM), while the Sorcin W105G mutant affinity for EGFR is an order of magnitude lower (*K*_D_ = 24 μM) (Fig. 7B), showing that the EF3 hand is important in EGFR recognition and binding. The same experiment, carried out in the absence of calcium, shows that wt Sorcin binds EGFR with decreased affinity (*K*_D_ > 20 μM, Supplementary Fig. 6B).

### Sorcin alters EGFR calcium signaling

In order to evaluate the effect of Sorcin on EGFR calcium signaling and regulation, luminescence-based measurements of cytosolic calcium were taken. We performed Ca^2+^ measurements using an organelle-targeted aequorin probe specific for the cytoplasm (cytAEQ). Cytosolic Ca^2+^ transients of H1299 cells transfected with either a non-specific control siRNA or a specific siRNA against Sorcin were measured after their exposure to 100 ng/mL EGF; however, no differences were observed as reported in Fig. 7C. Since the cytosolic Ca^2+^ concentrations reached after the stimulation with EGF were particularly low (< 0.8 μM), we decided to perform the same experiment using Histamine 100 μM as stimulus, in order to rule out the possibility that the administration of EGF alone was not sufficient to elicit a proper mobilization of Ca^2+^ in H1299 cells. As shown in Fig. 7D, the administration of Histamine resulted in comparable cytosolic Ca^2+^ transients, that remained unaltered upon Sorcin silencing, suggesting that the low Ca^2+^ levels observed after EGF administration were not dependent on the specific stimulation. To better characterize H1299 cells, we decided to perform the experiment using a mix of different stimuli, and even in this condition the Ca^2+^ transients were rather low (about 1 μM) (Fig. 7E), suggesting that H1299 cells can mobilize a restricted pool of Ca^2+^ in response to stimulation. Finally, to specifically focus on Sorcin roles on ER Ca^2+^ maintenance, we performed the experiment stimulating with EGF in the presence of both 2 mM EGTA, to prevent the influx of extracellular calcium, and 20 μM CPA, to inhibit SERCA pumps and the reuptake of calcium in the ER. With this experimental setting, we specifically measure ER Ca^2+^ release in response to EGF stimulation. Due to the very low Ca^2+^ peaks reached in H1299 cells (see above), we performed this experiment on HeLa cells: in this cell type, ER Ca^2+^ release after EGF stimulation was significantly decreased upon Sorcin silencing by about 17% with respect to the control, as shown in Fig. 7F. Therefore, Sorcin is involved in EGFR calcium signaling and regulation, with its silencing decreasing ER Ca^2+^ release in response to EGF; these data support Sorcin role in EGFR phosphorylation, signaling and proteostasis.

## Discussion

Calcium is long since considered important in EGFR internalization, EGFR signaling and EGFR proteostasis [14, 18, 64, 65]. However, the mechanisms that link calcium homeostasis to EGFR signaling in physiological and pathological conditions are not well-defined.

The present work was prompted by the correlation observed between the overexpression of Sorcin, an important calcium sensor oncoprotein, and cell migration, invasion, EMT and metastatic dissemination linked to of the PI3K/Akt/mTOR pathway [33, 66, 67]. Bioinformatic analysis carried out on the TCGA PanCancer study (cBioPortal) and using the Cancer Proteogenomic Data Analysis Site (cProSite), highlighted that Sorcin and EGFR overexpression are significantly correlated in cancer patients and associated with reduced overall survival in various cancer types, suggesting that these proteins could be functionally linked in cancer.

We then analyzed whether Sorcin knock-out affects EGFR expression, showing that in the bronchiolar region of the lungs EGFR expression is strongly reduced in Sorcin KO mice with respect to WT mice. We therefore studied the role of Sorcin in EGFR signaling processes, and in promoting cell proliferation, migration and invasion in cancer, and demonstrated that Sorcin acts at different levels on such processes, with a complex resulting effect. In physiological EGFR signaling (short time, low concentration EGF treatments), Sorcin silencing increases EGFR phosphorylation and decreases its signaling, acting on one of the main EGFR-dependent pathways, i.e., the RAS-RAF-MEK-ERK axis [68]. These pathways have already been associated with Sorcin in calcium-mediated angiogenesis, since Sorcin silencing in endometrial epithelial cells alters calcium homeostasis and reduces the expression of VEGF, PI3K and AKT, AKT phosphorylation and activation of NOS [37]. In colorectal cancer, Sorcin promotes metastasis and EMT via activation of PI3K/AKT signaling [63], while Sorcin silencing increases PTEN levels and decreases the expression of ABCB1, Bcl-2, Survivin, phosphorylated AKT and NF-kB, downstream to PI3K/AKT in nasopharyngeal carcinoma [33], in myeloma [69] and in human lung cancer A549/DDP cells [46]. On the other side, Sorcin silencing increases phosphorylation of ERK1/2 protein in HEI-OC1 cells and in leukemia cells [70].

Here, we show that Sorcin controls EGFR signaling and proteostasis and activates the RAS/ERK signaling cascade, participating in the regulation of migration and invasion.

The effect of Sorcin on the EGFR pathways is both direct and indirect. Sorcin interacts with EGFR in a calcium-dependent fashion, possibly via its EF3 hand, which is responsible for Sorcin activation, since binding of Ca^2+^ ion induces a large conformational change leading to the exposure of hydrophobic pockets that can engage its binding partners [24–26, 63]. Such interaction, as for the interaction with calmodulin, may favor EGFR internalization, tyrosine kinase activation and EGFR signaling [17]. In addition, since Sorcin can interact with IP3R [38] and modulates calcium fluxes through plasma membrane and ER [25, 28–31, 71–73], Sorcin regulates ion storage in ER and mitochondria, thereby acting on ER stress [22, 74–76].

Overall, Sorcin promotes cell proliferation, migration and invasion in cancer, while increasing resistance to chemotherapeutic agents. NSCLC represents about 80% of all lung cancers and is the leading cause of cancer deaths worldwide [77]. Erlotinib-based EGFR-targeted therapy is among the most important treatments available against NSCLC, both as a monotherapy and in combination with gemcitabine. However, the anticancer activity of erlotinib is limited by the development of resistance, which represents a challenge to clinicians and patients: increasing the effect of EGFR-targeted therapies is therefore a major goal [78–80]. Here we show that decreased Sorcin activity leads to reduced cell migration, invasion and EMT, via reduced RAS/ERK signaling, synergistically with erlotinib: strategies aimed at lowering Sorcin activity may prove important to formulate novel combination therapies with erlotinib and other EGFR-targeted therapies.

## Conclusions

The present work elucidates an important mechanism that links calcium homeostasis to EGFR signaling in physiological and pathological conditions. Sorcin regulates different features of EGFR-dependent signaling: Sorcin binds EGFR in a calcium-dependent fashion, regulates ER Ca^2+^ release after EGF stimulation, controls EGFR signaling and activates the RAS/ERK signaling cascade, participating in the regulation of migration and invasion. Sorcin silencing decreases cell migration, invasion and EMT, synergistically with EGFR inhibitors.

## Supplementary Information

Below is the link to the electronic supplementary material.

Supplementary file1 (PDF 464 KB)

Supplementary file2 (PDF 580 KB)

Supplementary file3 (PDF 1515 KB)

Supplementary file4 (PDF 994 KB)

Supplementary file5 (PDF 500 KB)

Supplementary file6 (PDF 180 KB)

## Data Availability

The data that support the findings of this study are available from the corresponding author upon reasonable request.

## Abbreviations

EGFR: Epidermal growth factor receptor
EGF: Epidermal growth factor
EMT: Epithelial–mesenchymal transition
CME: Clathrin-mediated endocytosis
NCE: Non-clathrin-mediated endocytosis
ESCRT: Endosomal sorting complex required for transport
PI3K: Phosphoinositide 3-kinase
TKIs: Small molecule tyrosine kinase inhibitors
VGCCs: Regulating voltage-gated calcium channels
PMCA: Plasma membrane Ca^2+^-ATPase
NMDARs: *N*-Methyl-d-aspartate receptors
NCX: Na^+^- Ca^2+^ exchanger
GRB2: Growth factor receptor-bound protein 2
NSCLC: Non-small cell lung cancer
SRI: Sorcin (SOluble Resistance-related Calcium binding proteIN)
MDR: Multidrug resistant
ER: Endoplasmic reticulum
RyRs: Ryanodine receptors
SERCA: Sarco(endo)plasmic Reticulum Ca^2+^-ATPase
UPR: Unfolded protein response
DsiRNAs: Dicer-substrate short interfering RNAs
GBM: Glioblastoma multiforme
CRC: Colorectal cancer
LUAD: Lung adenocarcinoma
OVCA: High-grade serous ovarian cancer
TCGA: Cancer genome atlas
NC: Negative control
GM130: Golgi matrix protein GM130
CHD2: N-Cadherin
TG: Thapsigargin
EGTA: Egtazic acid
CHX: Cycloheximide
