# Supplementary material for: Sorcin promotes migration in cancer and regulates the EGF-dependent EGFR signaling pathways
Source: Cell Mol Life Sci. 2023 Jul 13;80(8):202. doi: 10.1007/s00018-023-04850-4 (PMC10345051; doi:10.1007/s00018-023-04850-4)
Supplement: Supplementary file 1 — Supplementary file1 (PDF 464 KB) [file 18_2023_4850_MOESM1_ESM.pdf]

SRI/EGFR signature (RNAseq)

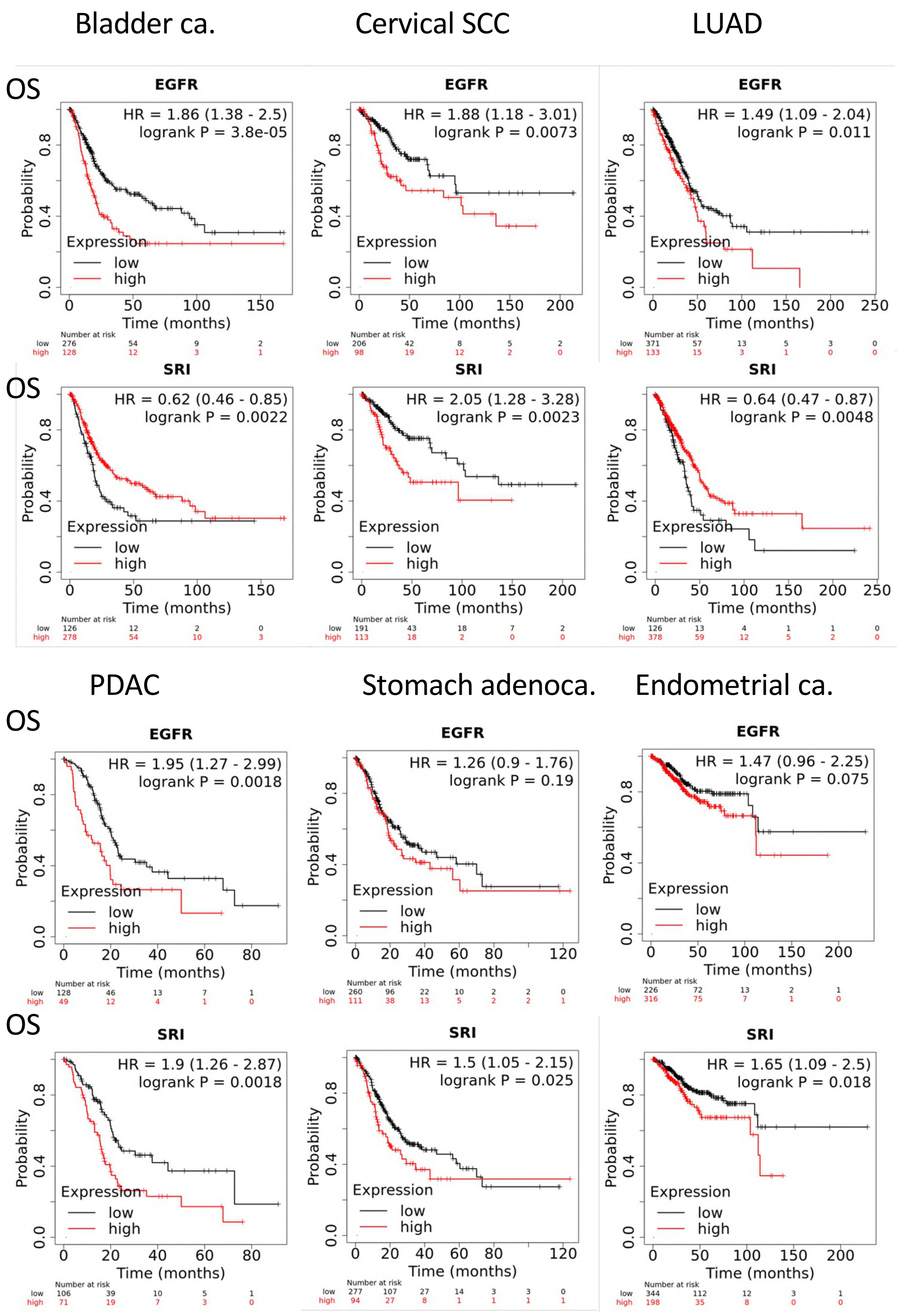

Supplementary Figure 1:

Kaplan-Meier analysis of the impact of EGFR or SRI expression (RNAseq) on overall survival in the indicated tumor types.
