# Supplementary material for: Sorcin promotes migration in cancer and regulates the EGF-dependent EGFR signaling pathways
Source: Cell Mol Life Sci. 2023 Jul 13;80(8):202. doi: 10.1007/s00018-023-04850-4 (PMC10345051; doi:10.1007/s00018-023-04850-4)
Supplement: Supplementary file 2 — Supplementary file2 (PDF 580 KB) [file 18_2023_4850_MOESM2_ESM.pdf]

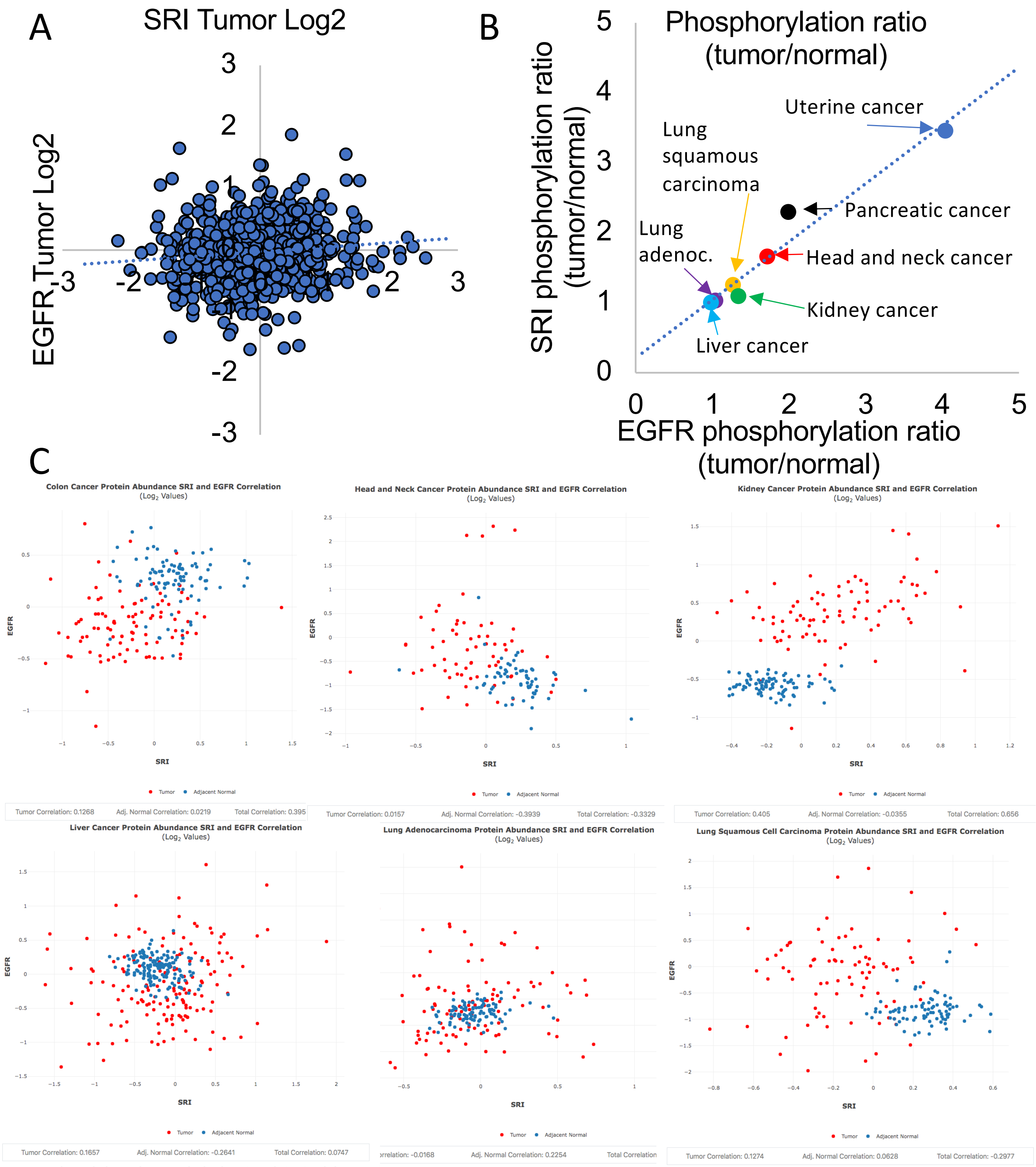

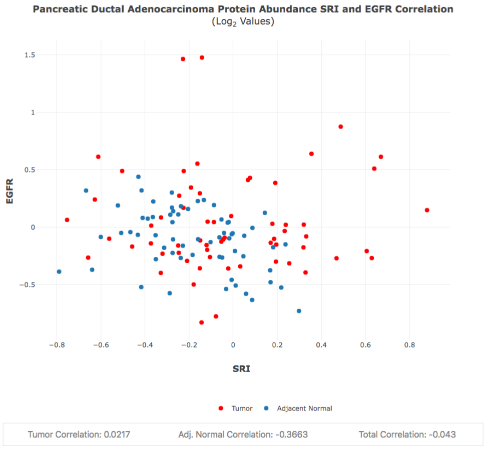

Supplementary Figure 2

cProSite analysis of correlation of Sorcin and EGFR protein expression and phosphorylation.

A: Expression of Sorcin and EGFR at protein level (correlation= +0.12), pooling all tumor data in the cProSite database.

B: Average increase of phosphorylation in tumors (phosphorylation in each tumor/ phosphorylation in normal surrounding tissue ratio) was obtained for 7 types of tumor for EGFR and SRI (correlation > +0.9).

C: Expression for EGFR and SRI for tumors evaluated individually. Positive correlation was obtained for 6 out of 7 tumors with at least 30 patients with data on abundance in tumors and adjacent normal tissue.
