# Supplementary material for: Sorcin promotes migration in cancer and regulates the EGF-dependent EGFR signaling pathways
Source: Cell Mol Life Sci. 2023 Jul 13;80(8):202. doi: 10.1007/s00018-023-04850-4 (PMC10345051; doi:10.1007/s00018-023-04850-4)
Supplement: Supplementary file 3 — Supplementary file3 (PDF 1515 KB) [file 18_2023_4850_MOESM3_ESM.pdf]

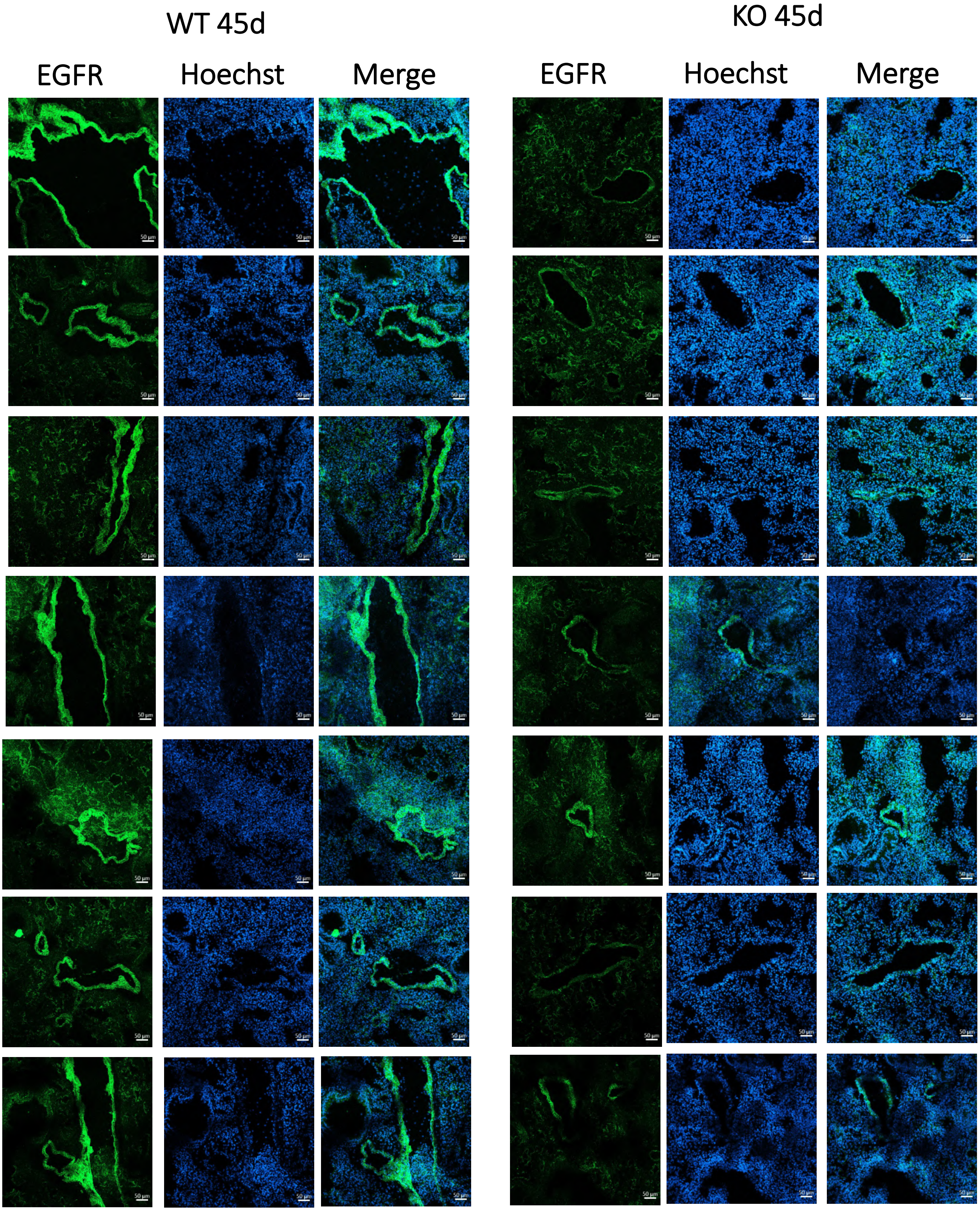

Supplementary Figure 3

Confocal microscopy analysis in lung tissues of adult (6-weeks old) C57/BL6 WT and KO Sorcin mice to evaluate the expression of EGFR protein (green). Scale bars, 50µm. Representative images of 10 lung's section per 4 mice per group (C57/BL6 WT versus KO Sorcin mice)
