# Supplementary material for: Sorcin promotes migration in cancer and regulates the EGF-dependent EGFR signaling pathways
Source: Cell Mol Life Sci. 2023 Jul 13;80(8):202. doi: 10.1007/s00018-023-04850-4 (PMC10345051; doi:10.1007/s00018-023-04850-4)
Supplement: Supplementary file 4 — Supplementary file4 (PDF 994 KB) [file 18_2023_4850_MOESM4_ESM.pdf]

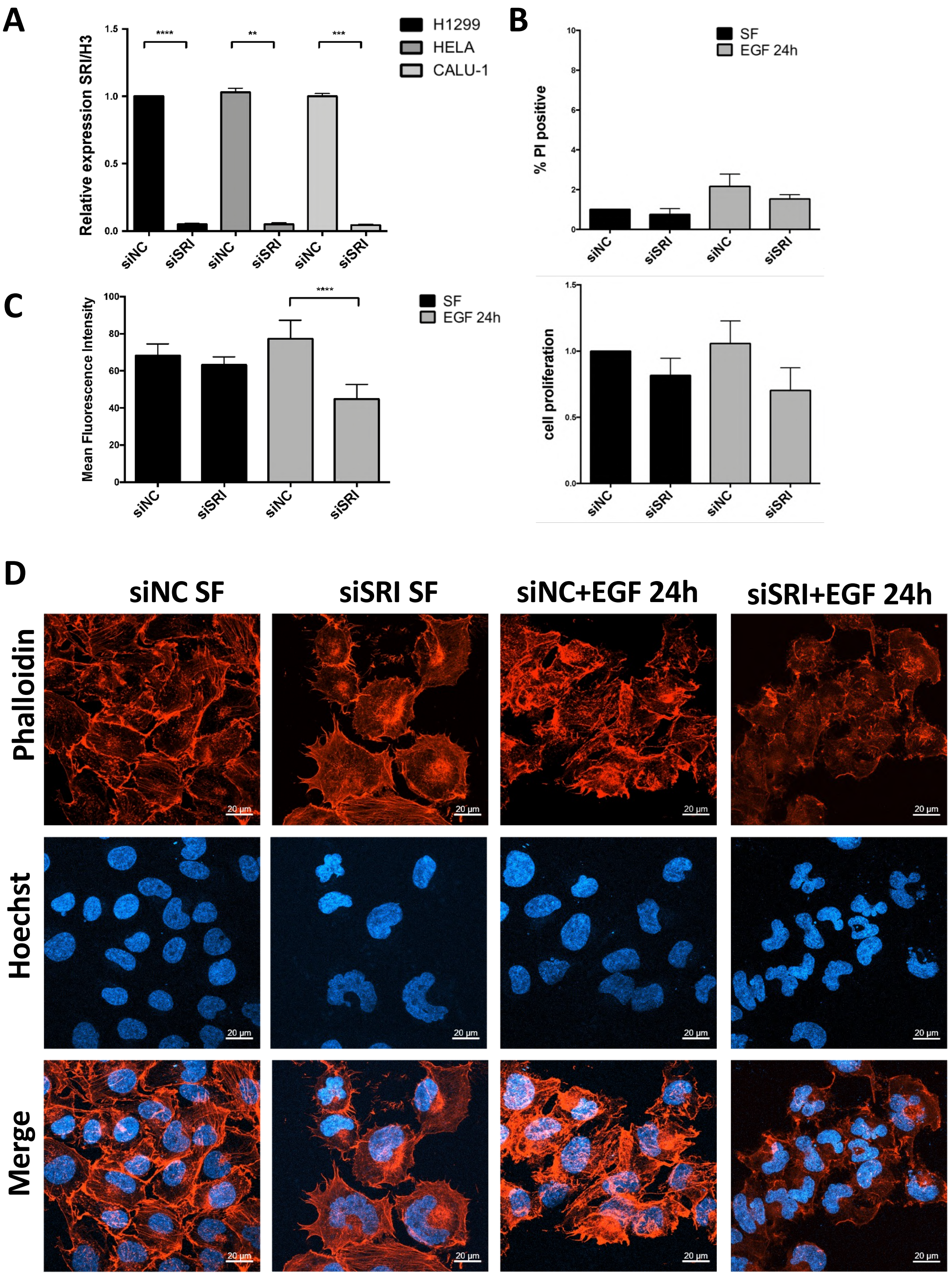


**Supplementary Figure 4**

A) Quantitative reverse transcription-PCR (qRT-PCR) of Sorcin (SRI) in H1299, HeLa and Calu-1 cells upon 48h of siRNA transfection. Error bars indicate means ± SEM. **p <0.01 ***p<0.001 and ****p < 0.0001 as determined by Student's t test.

B) Analysis of cell death and cell proliferation upon 48h of siRNA transfection, 2h of starvation in serum free medium (SF) and 24h of EGF treatment. Error bars indicate means ± SEM (n=3)

C) Quantification analysis of phalloidin fluorescence intensity by ImageJ software plugin (left panel). Error bars indicate means ± SEM. ****p < 0.0001 as determined by Student's t test (n=3).

D) Confocal microscopy analysis of phalloidin staining to evaluate the structure of the cytoskeleton in fixed cells, upon 48h of Sorcin silencing (siSRI), 2h of starvation in serum free medium (SF) and 24h of EGF treatment. Representative images of an experiment. Scale bars, 20μm.
