# Supplementary material for: Sorcin promotes migration in cancer and regulates the EGF-dependent EGFR signaling pathways
Source: Cell Mol Life Sci. 2023 Jul 13;80(8):202. doi: 10.1007/s00018-023-04850-4 (PMC10345051; doi:10.1007/s00018-023-04850-4)
Supplement: Supplementary file 5 — Supplementary file5 (PDF 500 KB) [file 18_2023_4850_MOESM5_ESM.pdf]

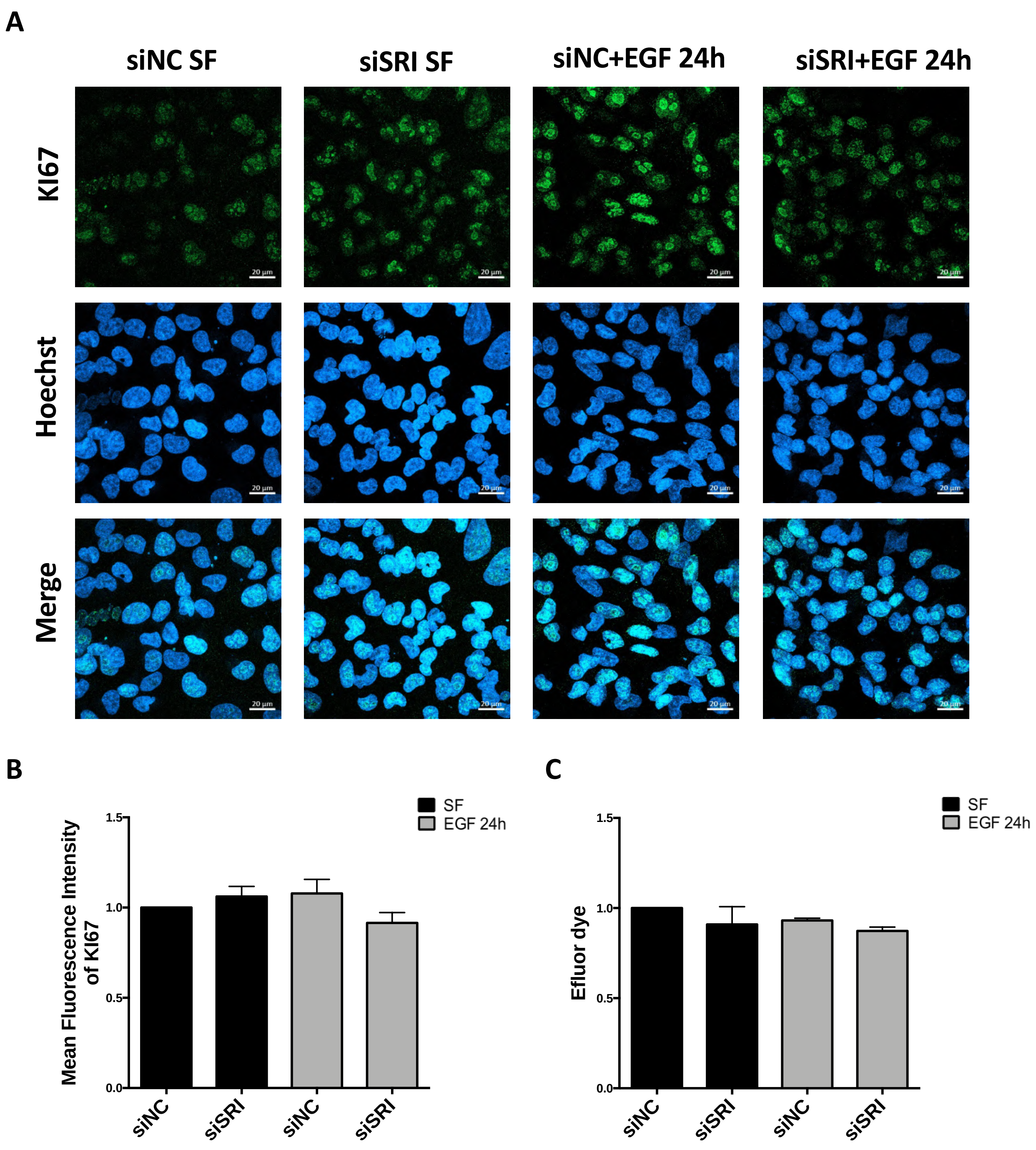

Supplementary Figure 5

A) Confocal microscopy analysis of KI67 protein to evaluate cellular proliferation, upon 48h of Sorcin silencing (siSRI), 2h of starvation in serum free medium (SF) and 24h of EGF treatment. Representative images of an experiment. Scale bars, 20µm.

B) Quantification analysis of KI67 fluorescence intensity by ImageJ software plugin (n=3).

C) Efluor dye analysis to measure cellular proliferation, upon 48h of Sorcin silencing (siSRI), 2h of starvation in serum free medium (SF) and 24h of EGF treatment (n=3).
