# Supplementary material for: Sorcin promotes migration in cancer and regulates the EGF-dependent EGFR signaling pathways
Source: Cell Mol Life Sci. 2023 Jul 13;80(8):202. doi: 10.1007/s00018-023-04850-4 (PMC10345051; doi:10.1007/s00018-023-04850-4)
Supplement: Supplementary file 6 — Supplementary file6 (PDF 180 KB) [file 18_2023_4850_MOESM6_ESM.pdf]

A

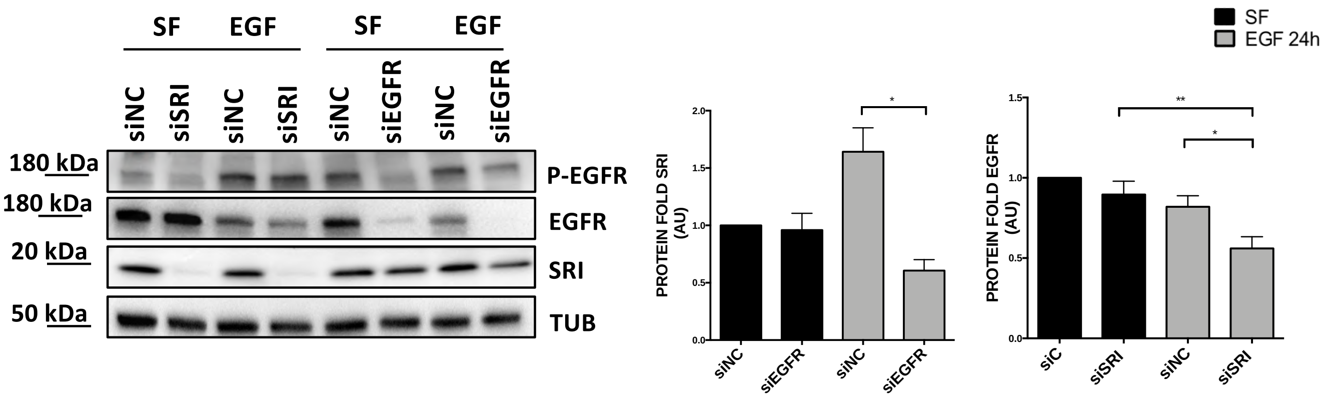

B

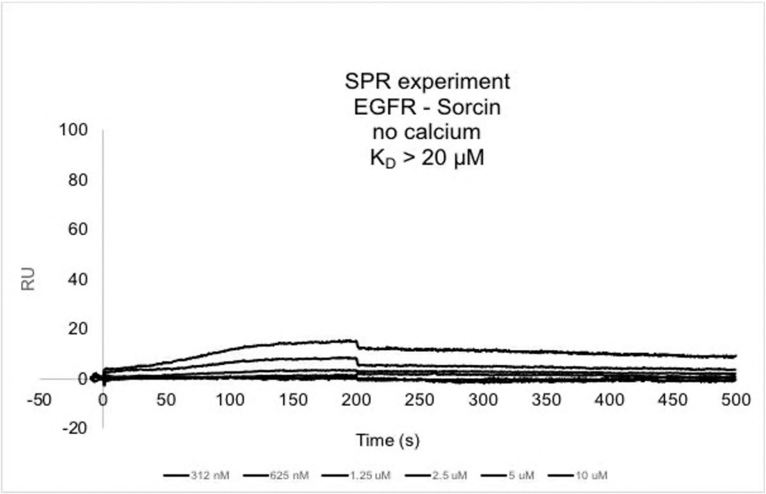

Supplementary Figure 6

A) H1299 cells were silenced for SRI and EGFR protein (siEGFR cells) for 48h, starved for 2h in serum free medium (SF) and treated with low doses of EGF for 24h (5ng/mL). Western blot analysis showed level expression of SRI, p-EGFR and EGFR. Densitometry analysis is shown in right graphs. Error bars indicate means ± SEM. *p<0.05 and **p<0.01 as determined by Student's t test (n=4).

B) SPR experiments showing the binding of wt Sorcin to immobilized C-terminal intracellular domain of EGFR. Sorcin was injected at the following concentrations: 312 nM, 625 nM, 1.25 µM, 2.5 µM, 5 µM and 10 µM, at a constant flow (nominal flow rate = 30 µl/min), in the absence of $CaCl_2$; EGFR binds wt Sorcin with a lower affinity (KD>20 µM) with respect to the same experiment carried out in the presence of calcium.
